# Physicochemical, Phytochemical and Sensory Properties of Myrobalan (*Prunus cerasifera* L.) Fruit Leather: Effects of Sugar Concentration and Enrichment with Blackcurrant and Bilberry Pomace Powders

**DOI:** 10.3390/foods14203457

**Published:** 2025-10-10

**Authors:** Cristina Paula Săpoi (Gheorghe), Alexandru Radu Corbu, Liliana Ceclu, Violeta Nour

**Affiliations:** 1 Faculty of Food Science and Engineering, “Dunărea de Jos” University of Galati, Domnească Street 111, 800201 Galati, Romania; cristina.gheorghe_2005@yahoo.com; 2 Department of Horticulture & Food Science, University of Craiova, 13 AI Cuza Street, 200585 Craiova, Romania; alexandru.corbu@edu.ucv.ro; 3 Faculty of Economics, Engineering and Applied Sciences, Cahul State University “B.P. Hasdeu”, Piaţa Independenţei, 1, 3900 Cahul, Moldova; ceclu.liliana@gmail.com

**Keywords:** fruit leather, berry by-products, color, phenolic compound, organic acids, antioxidant activity

## Abstract

Myrobalan plum is a widespread but underutilized fruit, rich in dietary fiber, organic acids and bioactive compounds. The present research was carried out to develop myrobalan plum leathers using different levels of sugar addition, and to improve their functionality by adding blackcurrant (BCP) and bilberry (BBP) pomace powders. The resulting fruit leathers were analyzed for color, titratable acidity, total phenolic content, antioxidant activity, organic acid profile, phenolic profile and sensory properties. Five samples were manufactured with different fruit pulp/sugar ratios of 100:0, 90:10, 80:20, 70:30 and 60:40, respectively. The myrobalan leathers prepared with 90% pulp and 10% sugar showed the highest sensory scores and physicochemical properties. BCP and BBP were added at 1% and 2% to the leather formulation manufactured at a myrobalan puree/sugar ratio of 90:10. Total phenolic content increased 2 and 3.5 times as a result of 1% and 2% BBP addition and only 1.15 and 1.29 times as a result of 1% and 2% BCP addition, respectively. Among the quantified phenolic compounds, epicatechin dominated in control myrobalan fruit leather, followed by catechin hydrate and chlorogenic acid. This research highlights the potential of processing myrobalan plums into fruit leathers, a nutritious and functional snack food, and of enhancing the product’s functional profile and sensory appeal by adding blackcurrant and bilberry pomace powders, thus contributing to the sustainable use of these by-products.

## 1. Introduction

Native to central and eastern Europe, as well as southwest and central Asia, *Prunus cerasifera* Ehrh, commonly known as the myrobalan or cherry plum, is one of the oldest and most widely distributed fruit species [1]. Growing in both wild and cultivated forms across neighboring regions of Europe and Asia [2,3], cherry plum is known for its easy adaptability to a wide variety of sites and for the ecosystem services it provides, including carbon sequestration and heavy metal accumulation, food for humans and animals and a source of nectar for pollinators, habitats for insects and nesting birds, biodiversity and climate change and esthetics of the space, landscaping and design [1]. In addition, cherry plum is recognized for its resistance to several plum diseases and for its use as rootstock in various fruit species production (plum, apricot, more rarely for peach) due to its ability to produce adventitious roots [2,4,5]. Having a characteristic sweet–sour taste, myrobalan plums are very popular plum-like edible fruits, often consumed not fully ripe in early spring [6,7]. Various shapes (round, flat-round or ovoid), sizes and colors (yellow, red, purple or almost black) of the fruit, as well as different shapes of the seed, characterize the different varieties of myrobalan plum [8]. The fruits are highly valued by consumers for their taste and early ripening time, or in relation with the gastronomic traditions; however, the fruits show a high diversity in morphological, nutritional and functional properties.

The myrobalan (cherry) plum (*Prunus cerasifera*) genotypes are also widely spread in the Oltenia region (southwestern Romania), from the plains to the hills, and are currently used in breeding due to their high resistance to extreme temperatures and diseases, adaptability to climate change, larger and more stable yields and early fruiting and ripening [1,9,10].

In recent years, the food industry is challenged to produce sustainable, functional products to meet the growing interest of consumers in adopting healthier diets [11,12]. One essential component of a healthy diet is the adequate consumption of fruits and fruit products as they are rich sources of essential nutrients for the human body [13]. The “formed fruit” such as leathers, jerky, rolls or twists, have become popular in our days and are considered nutritious and healthy snacks, a healthier alternative to candies and confections, with a nutrient density similar to fruit-based bars but higher as compared to fresh fruit due to dehydration [14,15].

Fruit leathers, also called fruit pestils or fruit rolls, are processed by drying a thin layer of fruit pulp, puree or juice concentrate combined with sweeteners and other natural additives. The resulting “formed fruit” is sticky, chewy, has a pleasant appearance, soft texture and an appealing taste [16] and is a concentrated source of vitamins, minerals, dietary fibers and bioactive compounds [17,18]. Fruit leather is becoming popular also for its low-calorie content and prolonged shelf life without refrigeration requirements [19,20].

Many studies have aimed to develop fruit leathers from various fruits (apple, pear, guava, mango, strawberry, kiwifruit, pumpkin, pineapple, pomegranate) [21,22,23,24,25,26] and to investigate the effects of some process parameters (drying air temperature, sample thickness) and ingredients (hydrocolloids, sugars and additives) on their drying process, physicochemical and phytochemical properties [24,25,26,27,28,29,30,31], but none have been reported for myrobalan plum leather. However, production of fruit leathers could add value to myrobalan fruits which are less acceptable for the fresh market.

Addition of other fruits or fruit by-products in the processing of fruit leather can be approached to obtain nutritional and functional benefits. Previously, Diamante et al. [22] explored the addition of blackcurrant concentrate to the apple fruit leather while Momchilova et al. [27] added dried chokeberries (*Aronia melanocarpa*) to plum (*Prunus domestica*) leathers. With a view to improve the phytochemical content and antioxidant activity of fruit leathers, Mphaphuli et al. [25] proposed the addition of natal plum (*Carissa macrocarpa*) to mango fruit leathers, while Torres et al. [32] enriched apple or quince leathers with maqui berry extract.

Recent studies demonstrated the potential of blackcurrant and bilberry pomaces in various food applications, including yogurt, cookies, corn-based extruded snacks and fruit leather production [26,33]. In a recent study, our research group demonstrated that addition of wild bilberry and blackcurrant pomace powders may be a promising strategy to improve the taste, flavor, bioactive content and antioxidant activity of pear leathers [26]. The combination of myrobalan fruit with berry pomaces, such as blackcurrant and bilberry pomaces, for producing fruit leather has not been explored. This study addresses this gap by developing myrobalan and myrobalan–berry pomace leathers, aiming to enhance nutritional value and promote sustainable myrobalan and berry by-products utilization. Moreover, this study examined the physicochemical, phytochemical and sensory attributes of the developed fruit leathers to evaluate their commercial potential.

## 2. Materials and Methods

### 2.1. Materials

Fully ripe myrobalan fruits (*Prunus cerasifera* L.) from the cultivar “Miroval”, uniform in size and shape, were harvested in July 2025 from an experimental collection orchard planted in 2016 in Cornu village (Dolj county, southwestern Romania). The fruits were transferred to a laboratory, washed properly with running tap water, drained and stored at −20 °C until processing.

Blackcurrants (*Ribes nigrum* L.) and bilberries (*Vaccinium myrtillus* L.) collected from the wild flora of Vâlcea county (Oltenia Region, southwestern Romania) were processed into juice at a fruit processing company from Vaideeni (Vâlcea county, Romania). The fresh pomaces obtained as by-products were dried at 57 °C in a laboratory dehydrator (Deca +SS Design, Profimatic, Cluj-Napoca, Romania), ground to a powder, sieved through a mesh screen (0.5 mm) and placed at 20 °C in the dark. Table sugar and lemons (“Verna” cultivar) were acquired from a local supermarket while pectin was from Sosa Ingredients S.L (Navarcles, Spain).

### 2.2. Chemicals

Gallic acid, Folin–Ciocalteu’s reagent, sodium acetate, 2,2-diphenyl−1-picrylhydrazyl (DPPH), 6-hydroxy-2,5,7,8-tetramethylchroman-2-carboxylic acid (Trolox), potassium dihydrogen phosphate, phosphoric acid, as well as standards of phenolic acids (caffeic, chlorogenic, ferulic, gallic, *p*-coumaric, syringic, *trans*-cinnamic, and vanillic) and flavonoids (catechin hydrate, epicatechin, quercetin and rutin) were purchased from Sigma-Aldrich (Steinheim, Germany). Anhydrous sodium carbonate, methanol (HPLC grade), citric, malic, tartaric, acetic, ascorbic and oxalic acids were from Merck (Darmstadt, Germany).

### 2.3. Preparation of Fruit Leathers

In the first experiment, myrobalan fruit leathers were prepared from myrobalan puree using different sugar addition levels. After defrosting myrobalan fruits, the peels and kernels were easily removed by passing the pulp through a sieve, and the myrobalan flesh was mashed in a blender (Bosch MS61B6170, 1000 W, Stuttgart, Germany) to obtain a homogeneous puree. The myrobalan puree and table sugar were mixed at five different ratios: 100:0 (MFLC—control), 90:10 (MFL10), 80:20 (MFL20), 70:30 (MFL30) and 60:40 (MFL40). Pectin (1%, *w*/*w*) and lemon juice (2.5%, *w*/*w*) were added in each formulation, and the mixtures were homogenized at high speed for 2 min to obtain smooth and homogeneous blends. Two batches of 500 g (myrobalan puree + sugar) blend were manufactured for each formulation on different days. Each blend was then poured as a uniform thin layer into a stainless-steel tray (covered with greaseproof paper greased with oil) and dried at 57 °C in a hot air cabinet dryer (Deca +SS Design, Profimatic, Cluj-Napoca, Romania). The moisture loss of the leathers was determined every 30 min during drying by weighing the tray using a digital scale with a 0.01 g accuracy (Radvag WLC/6/A2, Radom, Poland). The drying was carried out until a final moisture content below 15% was reached, calculated from weight loss and dry matter content data. After cooling, fruit leather samples were peeled off from the paper, cut into 3 × 2 cm pieces, rolled up in aluminum foil and stored at 4 ± 1 °C until analysis.

Moisture content was analyzed gravimetrically by drying the samples at 102 ± 2 °C in a laboratory oven to obtain a constant weight in three consecutive measurements. Moisture content was calculated as an average of three parallel determinations.

In a second experiment, blackcurrant (BCP) and bilberry (BBP) pomace powders were added at two different addition levels (1% and 2%) to the myrobalan leather formulation manufactured at a myrobalan puree/sugar ratio of 90:10 (MFL10). Five different formulations of fruit leather were manufactured in this experiment: MFL10 (control leather), MFL10BC1 and MFL10BC2 made by adding 1% and 2% BCP, respectively, and MFL10BB1 and MFL10BB2 made by adding 1% and 2% BBP, respectively.

### 2.4. Color Analysis and Titratable Acidity

The CIEL*a*b* color coordinates (L*—darkness/whiteness, a*—greenness/redness and b*—blueness/yellowness) of the fruit leathers were measured using a PCE-CSM1 reflectance colorimeter (PCE Instruments, Southampton, UK). Six measurements were performed at various points on each sample. Chroma (C) and hue angle (h) were calculated according to Equations (1) and (2), respectively:

(1) $$C\;=\;\sqrt{a^{*2}+b^{*2}}$$

(2) $$\left(h)=\tan^{-1}(b^{*}/a^{*}\right)$$

The color difference (ΔE) between the fruit leathers and the control was calculated according to Equation (3) while the browning index (BI) was calculated according to Equation (4) [29].

(3) $$∆E=\sqrt{{\left(L_{1}^{*}-L_{0}^{*}\right)}^{2}+{\left(a_{1}^{*}-a_{0}^{*}\right)}^{2}+{\left(b_{1}^{*}-b_{0}^{*}\right)}^{2}}$$

where $L_{1}^{*}$, $a_{1}^{*}$ and $b_{1}^{*}\;$represent color values of the fruit leathers and $L_{0}^{*}$, $a_{0}^{*}$ and $b_{0}^{*}\;$are color values of control leather.

(4) $$BI=\frac{100\times (X-0.31)}{0.17}$$

where $X=\frac{a^{*}+1.75L^{*}}{5.64L^{*}+a^{*}-0.301b^{*}}$.

Titratable acidity was estimated by titrating to pH 8.2 against 0.1 N NaOH an extract obtained from 10 g of fruit leather homogenized and diluted to 100 mL with distilled water [34]. Duplicate extracts were prepared for each sample with duplicate titration on each extract. The results were expressed as grams of malic acid (MA) per 100 g of fruit leather.

### 2.5. Preparation of Phenolic Extract

To prepare the methanolic extract, a 1 g sample of fruit leather was vortexed for 2 min with 10 mL of methanol, and the mixture was kept for 60 min in a Bandelin Sonorex Digital 10P ultrasonic bath (Bandelin Electronic GmbH, Berlin, Germany). It was then centrifuged at 2500 × *g* for 5 min, filtered through Whatman no. 1 filter paper and stored at 2–4 °C until subsequent analysis.

### 2.6. Total Phenolic Content

The Folin–Ciocalteu procedure reported by Singleton et al. [35] was carried out to assess the total phenolic content (TPC) in the fruit leathers. Aliquots of 0.1 mL extract were fused with 6 mL distilled water and 0.5 mL aqueous solution of Folin–Ciocalteu reagent (1:1 *v*/*v*). After 3 min, 1.5 mL of 20% (*w*/*v*) Na_2_CO_3_ solution and 1.9 mL of distilled water were added. The resulting solution was stirred in a vortex and allowed to stand in the dark for 30 min at 40 °C, then the absorbance was recorded at 765 nm using a Varian Cary 50 UV spectrophotometer (Varian Co., Cary, NC, USA). The results were expressed as milligrams of GAE (gallic acid equivalents) per 100 g of fruit leather. The analysis was repeated three times for each sample.

### 2.7. DPPH Radical Scavenging Activity

DPPH radical scavenging activity (RSA) was determined in the extracts using the spectrophotometric method presented by Oliveira et al. [36]. Briefly, aliquots of 50 μL extract were mixed with 3 mL of 0.004% DPPH solution. After staying in the dark for 30 min, the absorbance was recorded at 517 nm wavelength using a Varian Cary 50 UV spectrophotometer (Varian Co., USA). Finally, the DPPH radical scavenging activity was determined according to Equation (5):

DPPH scavenging activity (%) = [1 − Ac/As] × 100(5)

where Ac and As represent the absorbance of the control and test sample, respectively.

The results were expressed in millimoles Trolox per 100 g fruit leather.

### 2.8. Chromatographic Analysis of Organic Acids

The organic acids were detected and quantified in the aqueous extract of fruit leathers by high-performance liquid chromatography according to the method developed by Nour et al. [37] using a Finnigan Surveyor Plus HPLC system (Thermo Electron Corporation, San Jose, CA, USA). For the extraction of organic acids, samples of fruit leather (2 g) were homogenized with 30 mL of distilled water, then the mixtures were centrifuged at 6000 rpm for 10 min. The supernatants were injected after filtration through a nylon syringe filter (0.45 μm). The compounds were separated on a Hypersil Gold aQ column (5 μm, 250 × 4.6 mm) in isocratic conditions at 10 °C using a 50 mM KH_2_PO_4_ aqueous solution (pH 2.8) as the mobile phase, eluted at a flow rate of 0.7 mL/min. The diode array detector was set at λ = 254 nm for ascorbic acid and λ = 214 nm for malic, citric, tartaric and oxalic acids. Concentration of organic acids was expressed in mg per 100 g of fruit leather.

### 2.9. Chromatographic Analysis of Phenolic Compounds

High-performance liquid chromatography was used to detect and quantify phenolic compounds in the methanolic extracts of fruit leathers, according to the method previously described by Nour et al. [38]. The assay was performed with a Finnigan Surveyor Plus HPLC system (Thermo Electron Corporation, San Jose, CA, USA) equipped with a diode array detector. Separation was achieved in 60 min at 20 °C on a reversed-phase Hypersil Gold C18 column (250 mm × 4.6 mm, 5 μm) provided by Thermo Electron Corporation (San Jose, CA, USA). Elution was performed at 1 mL/min flow rate, using a mixture of 1% aqueous acetic acid (A) and methanol (B) as the mobile phase. The following gradient was used for elution: 90% A and 10% B as initial conditions, linear gradient from 90% A to 80% A in 20 min, linear gradient from 80% A to 60% A in 7 min, 60% A for 25 min, linear gradient from 60% A to 80% A in 5 min and linear gradient from 80% A to 90% A in 3 min. Detection was performed at 254, 278 and 300 nm, according to absorption maxima of the phenolic compounds. Identification was performed based on retention time of standards while quantification was achieved according to the peak area measurements. Concentration of phenolic compounds was expressed in mg per 100 g of fruit leather.

### 2.10. Sensory Analysis

Fruit leathers were sensory evaluated one day after manufacture. A sensory panel of 24 members comprising staff and students from the Department of Horticulture and Food Science from University of Craiova was selected to evaluate the appearance, color, flavor, taste, texture and general acceptability of fruit leathers. The sensory trial was conducted in two sessions, the first on the effects of sugar addition on the sensory attributes of myrobalan leathers (evaluation of MFLC, MFL10, MFL20, MFL30 and MFL40 samples) and the second session on the enrichment of the myrobalan leathers with blackcurrant and bilberry pomace powders (evaluation of MFL10, MFL10BC1, MFL10BC2, MFL10BB1, MFL10BB2 samples). The trials were carried out at 20 °C in a room with proper daylighting using the 9-point hedonic scale varying from 9 “like extremely” to 1 “dislike extremely”. The assessment was performed in random order within each session and water was provided to the panelists to rinse their mouth between evaluations. This study was approved by the Committee on Ethics of University of Craiova (Craiova, Romania) based on the informed consent from the participants. Two radar graphs presenting the sensory profile were plotted based on the results.

### 2.11. Statistical Analysis

Statgraphics Centurion XVI (StatPoint Technologies, Warrenton, VA, USA) was used to carry out the statistical analysis. All the physicochemical and phytochemical analysis were performed in triplicate and data were reported as mean values ± standard deviation. Analysis of variance (ANOVA), followed by the Least Significant Difference (LSD) multiple range test, was carried out to compare the means. The differences were considered statistically significant using a *p*-value threshold of 0.05.

## 3. Results and Discussion

### 3.1. Physicochemical and Phytochemical Properties of Myrobalan Fruits

The following results (presented as mean values ± standard deviation) were found on the physicochemical properties of myrobalan fruits used in the present study: weight (g) = 4.63 ± 0.26; volume (cm^3^) = 4.40 ± 0.24; stone weight (g) = 0.43 ± 0.05; pulp ratio (%) = 90.49 ± 1.38; moisture content (%) = 82.65 ± 0.39; soluble solids content (%) = 14.33 ± 0.47; titratable acidity (mg MA/100 g) = 3.14 ± 0.05. The results are in good agreement with those previously reported by Cosmulescu et al. [9] in myrobalan fruits from the semi-cultivated flora of Oltenia (southwestern Romania).

The results on TPC and RSA in the flesh and peel of myrobalan fruits as well as in the blackcurrant pomace powder (BCP) and bilberry pomace powder (BBP) used in the present study are presented in Table 1.

A TPC of 85.64 ± 2.77 and 138.36 ± 5.78 mg GAE/100 g was found in the flesh and peel of myrobalan fruits, respectively. In good agreement with our results, Sottile et al. [5] reported TPC in the range 34.52–97.63 mg GAE/100 g fw in a new accession of myrobalan (*Prunus cerasifera* L.) from Sicily (Italy) while Wang et al. [39] found TPC between 134 and 213 mg GAE/100 g fw in yellow myrobalan fruits. Dunaevskaya et al. [10] reported between 151.5 and 470 mg GAE/100 g in four cherry plum cultivars grown in Crimeea, while Gunduz and Saracoglu [6] found total phenolic content ranging from 13.68 to 58.31 mg GAE/100 g fw in eighteen *Prunus cerasifera* accessions from the Mediterranean region of Turkey. The mean values found for DPPH radical scavenging activity were 0.35 ± 0.02 and 0.44 ± 0.01 mmol Trolox/100 g for flesh and peel, respectively. In agreement with our results, Wang et al. [39] reported a mean antioxidant activity of 1.30 mmol/100 g fw in yellow myrobalan fruits. BCP and BBP, consisting mainly of skins and seeds of the corresponding berries, are very rich in phenolic compounds and their dehydrated state further concentrates these compounds. TPC of BBP was found 3.5 times higher as compared with BCP, while RSA was 4.1 times higher in BBP as compared with BCP.

### 3.2. Drying Process

**Figure 1:**
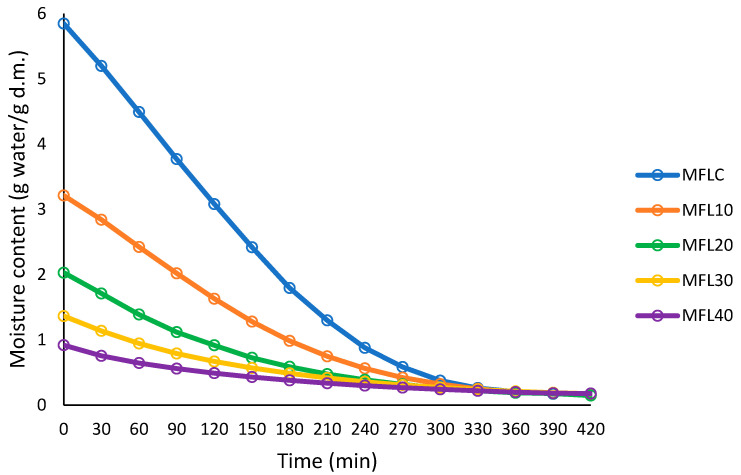
Drying curves at 57 °C of the myrobalan leathers made with different pulp/sugar ratios.

**Figure 2:**
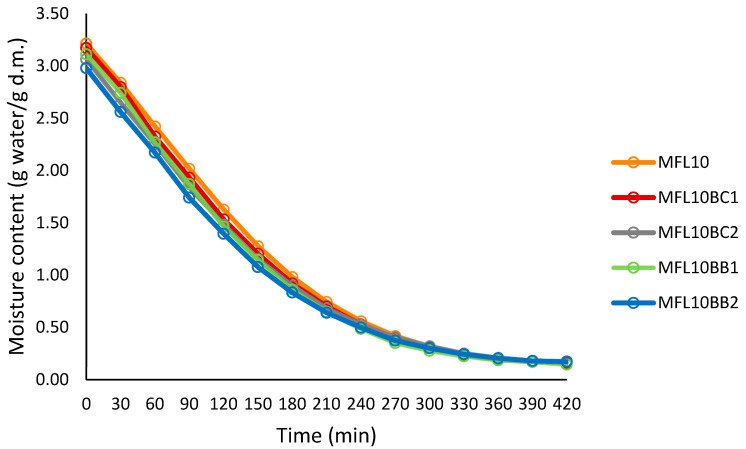
Drying curves at 57 °C of the control myrobalan leather (puree/sugar ratio = 90:10) and fruit leathers made with blackcurrant and bilberry powder addition.

The drying process of the fruit leathers at 57 °C was followed by plotting the sample moisture content (g water/g dry matter) against drying time, as presented in Figure 1 and Figure 2. Sugar addition had a major impact on the initial moisture content of the puree mixture; the higher the sugar addition level, the lower the initial moisture content. The increasing level of sugar resulted in a progressive decrease in the drying rate as compared with the control. The puree/sugar mixture with higher sugar level had lower initial moisture content (85.39% in MFLC, 76.25% in MFL10 and 47.85% in MFL40).

The decrease in drying rate as the sugar level increased may be ascribed to the restriction of moisture diffusion by sugar which acts as a barrier to the migration of water during drying. Previous studies have demonstrated the higher stickiness and lower moisture diffusivity of the water in sugar-infused fruits as compared with the fresh fruits [40].

The final leather thickness increased as the sugar content increased (Table 1) as a result of the decrease in the amount of water removed during drying. The higher thickness of the leather may constitute another argument for reducing the drying rate of the samples with higher sugar content. Previous studies also found that sample thickness considerably affects the drying rate, which increased as the sample thickness decreased [17,24,41].

Addition of the berry pomace powders resulted in the decrease in the initial moisture content of the puree mixture due to their low moisture content (76.25% in MFL10, 75.38% in MFL10BC2 and 74.86% in MFL10BB2). The addition also reduced the drying rate in myrobalan fruit leathers as compared to the control due to the high water-holding capacity of the pomace powders, mainly as a result of their high fiber content. Gujral et al. [42] also reported the reduction in the drying rate of both pineapple and mango leather as a result of sucrose, pectin or maltodextrin addition.

### 3.3. Color and Titratable Acidity

The effects of the pulp/sugar ratio on color parameters and titratable acidity (TA) of myrobalan leathers are presented in Table 2. The lightness (L* values) increased with increasing sugar content but the variation became statistically significant (*p* < 0.05) above 20% sugar content. Redness (a* values) significantly decreased while hue angle and ΔE progressively increased with increasing sugar content. The values of hue angle (56.62–63.55) indicate that myrobalan leathers are closer to the yellow tone. The total color variation was 1.28 for the MFL10 sample and between 3.67 and 4.29 for the samples with higher sugar content. According to the guide on color perception based on the amount of ∆E values presented by Karma [43], these values means that the difference between MFL10 and the control is “perceptible through close observation” (∆E values between one and two), while the other samples fit the “perceptible at a glance” attribute, indicating that the difference between their color and that of the control is easily noticeable without needing to closely examine them.

As expected, titratable acidity significantly decreased as the sugar content increased. Titratable acidity was very high in the control myrobalan leather (10.98 g MA/100 g) and, although it decreased, it remained quite high even in the samples with high sugar addition (3.32 and 2.41 g MA/100 g in MFL30 and MFL40, respectively). Addai et al. [44] reported TA between 1.48% and 1.63% in papaya leathers, Nizamlioglu et al. [45] found TA values between 0.90% and 1.70% in fruit leathers from apple and plum, while Phuong et al. [46] reported TA between 1.29% and 1.36% in mulberry fruit leathers. A high acidity level protects the color and flavor and extends the product’s shelf life.

**Figure 3:**
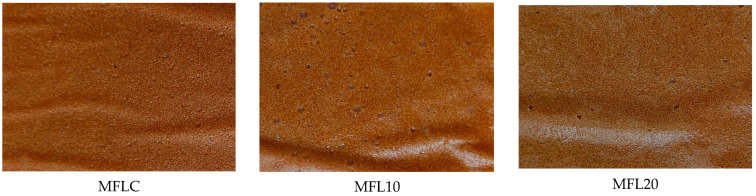

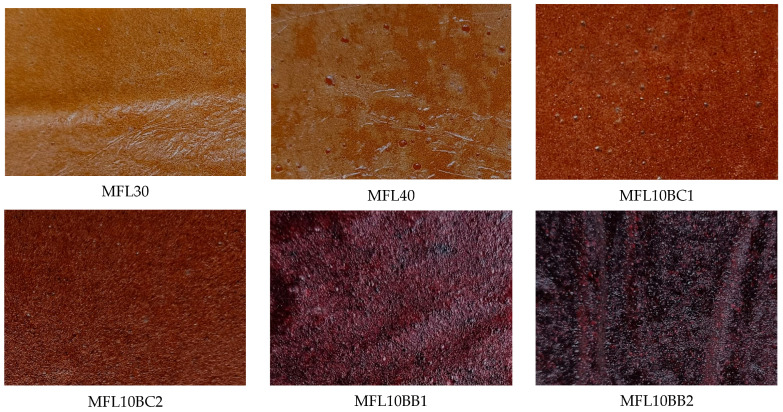
Appearance of the fruit leathers.

Table 3 presents the effects of blackcurrant (BCP) and bilberry (BBP) pomace powder addition on the physicochemical properties of myrobalan fruit leathers. All enriched formulations registered a significant decrease in lightness (L* values) as compared with the control. The a* and b* values decreased also as a result of both the enrichment with BCP and BBP. L*, a* and b* values were significantly (*p* < 0.05) lower in the formulations enriched with BBP than in those with BCP. Chroma values of 24.50 and 20.89 were found in myrobalan leathers enriched with 1% and 2% BCP, respectively, and only 11.16 and 7.36 in those enriched with 1% and 2% BBP, respectively. Chroma values between 3.93 and 19.86 were found by Diamante et al. [22] in fruit leathers made from apple and blackcurrant juice concentrates to which pectin was added. The appearance of the fruit leathers is shown in Figure 3.

### 3.4. Total Phenolic Content and DPPH Radical Scavenging Activity

**Figure 4:**
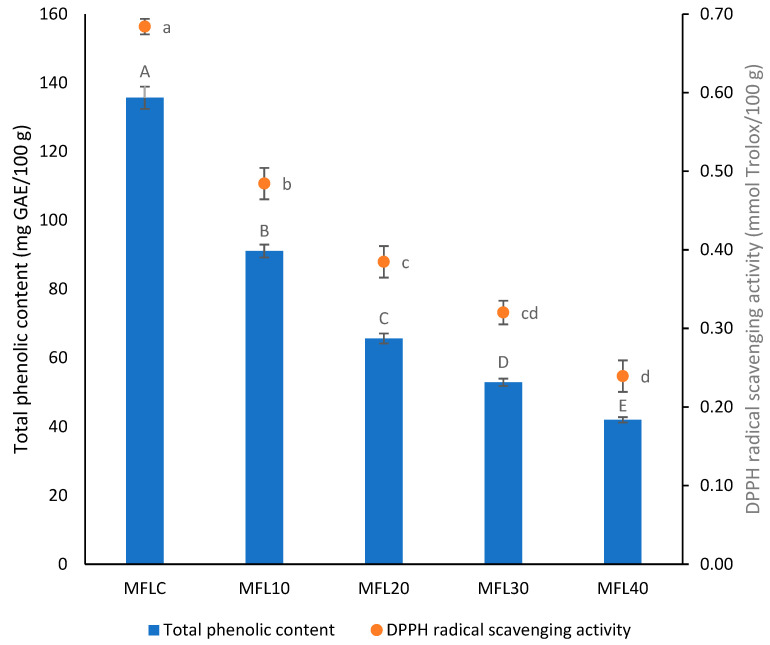
Effect of the pulp/sugar ratio on TPC and RSA of the myrobalan leather. Different lowercase letters indicate significant differences in RSA between fruit leather formulations (*p* < 0.05) while different uppercase letters indicate significant differences in TPC between fruit leather formulations.

The effect of the pulp/sugar ratio on TPC and RSA of the myrobalan leathers is illustrated in Figure 4. A mean TPC of 135.64 mg GAE/100 g was found in the control myrobalan leather. Consistent with our findings, Nizamlioglu et al. [45] found between 70.9 and 267.3 mg GAE/100 g in plum and apple–plum leathers while Addai et al. [44] reported TPC of 104.71 and 121.4 mg GAE/100 g in papaya leathers. Much higher values were found previously in mulberry fruit leathers (866–1213 mg GAE/100 g dw) [46] or in pomegranate fruit leathers (742.4–868.8 mg GAE/100 g dw) [24].

A low RSA was found in the control myrobalan leather (0.53 mmol Trolox/100 g). Previously, Kamiloglu and Capanoglu [47] reported the antioxidant activity of the plum pestil as 1.14 g TEAC/100 g dm. Comparatively, Quintero Ruiz et al. [48] reported RSA between 1.7 and 3.3 mmol TE/100 g in rose hip leathers, Tontul and Topuz [28] found 1.24–2.00 g TEAC/100 g dw in pomegranate pestils, while Chen and Martynenko [49] found 5.91–7.19 mmol TE/100 g dw in blueberry leathers. The effect of sugar addition on the total phenolic content in leather was found to be significant (*p* < 0.05). Expectedly, both total phenolic content and DPPH radical scavenging activity progressively decreased as the sugar addition level increased. Other previous studies reported a similar evolution of the antioxidant content in leathers with increasing sugar addition level [20,30].

**Figure 5:**
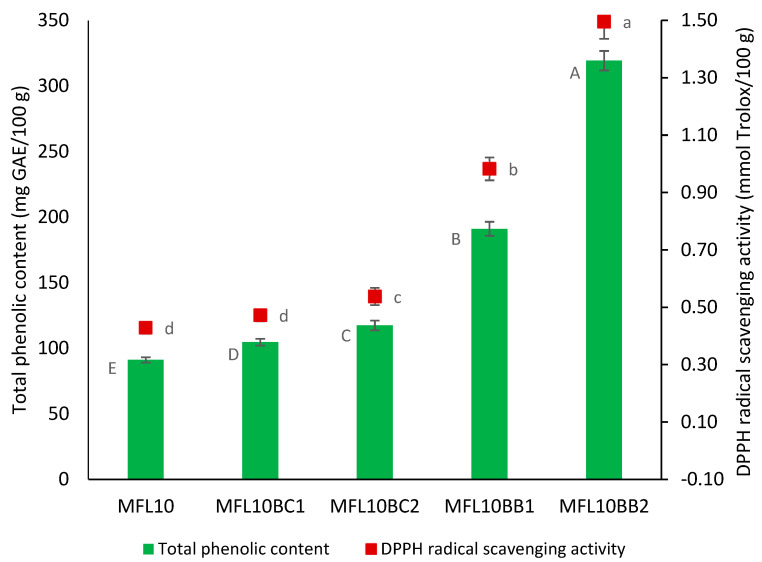
Effect of blackcurrant and bilberry pomace powder addition on TPC and RSA of the myrobalan fruit leather. Different lowercase letters indicate significant differences in RSA between fruit leather formulations (*p* < 0.05) while different uppercase letters indicate significant differences in TPC between fruit leather formulations.

The total phenolic content and antioxidant activity of myrobalan plum leathers enriched with BCP and BBP are presented in Figure 5. TPC in myrobalan leathers increased 2 and 3.5 times as a result of 1% and 2% bilberry pomace powder addition, respectively, reaching 191.05 and 319.27 mg GAE/100 g in MFL10BB1 and MFL10BB2, respectively. As a result, RSA increased 2.27 and 3.48 times, respectively. Lower multiplication rates of total phenolic content were recorded as a result of BCP addition (1.15 and 1.29 times in MFL10BC1 and MFL10BC2, respectively) as compared with BBP due to the lower total phenolic content in BCP as compared with BBP (Table 1). Previously, the addition of bilberry pomace powder determined the increase in the total phenolic content of pear leathers by 2.03, 3.26 and 4.45 times at addition levels of 0.5, 1.0 and 1.5%, respectively, reaching 310.64 mg GAE/100 g in pear leather with 1.5% bilberry pomace powder addition level. Enrichment of pear leathers with 1.5% BCP resulted in an increase by only 1.60 times of TPC (112.45 mg GAE/100 g) as compared with the control pear leather (70.45 mg GAE/100 g) [26].

### 3.5. Organic Acids

The organic acid profile of control myrobalan leather is dominated by malic acid (7.80 g/100 g), in agreement with previous studies stating malic acid as the prevalent acid in plums [50,51,52,53]. The increase in the pulp/sugar ratio resulted in the progressive and significant (*p* < 0.05) decrease in the content of all organic acids due to the dilution effect exerted by sugar (Table 4). Addition of BCP determined the significant increase in the citric acid content and the decrease in the oxalic acid content while tartaric acid content did not show significant variations (Table 5). The increase in citric acid content was to be expected considering that, in blackcurrants, citric acid represents more than 70% of the total content of simple acids [54,55]. Citric acid is also the main organic acid in bilberries (*Vaccinium myrtillus* L.) [56], therefore addition of BBP increased the citric acid content, but the growth was significant (*p* < 0.05) only at 2% BBP addition level. The ascorbic and malic acid contents were not significantly (*p* < 0.05) influenced by the addition of either BCP or BBP. The ascorbic acid content was low in all samples, probably as a consequence of its high instability and susceptibility to oxidation [30].

### 3.6. Phenolic Compounds

The phenolic content of the myrobalan leathers as affected by the pulp/sugar ratio is presented in Table 6 while Table 7 shows the effect of BCP and BBP addition on the phenolic content of myrobalan leathers. Among the quantified phenolic compounds, epicatechin dominated (5.37 mg/100 g) in control myrobalan fruit leather, followed by catechin hydrate (1.15 mg/100 g) and chlorogenic acid (0.47 mg/100 g). Addition of sugar exerted a diluting effect and, as a result, the content of all phenolic compounds progressively decreased as the sugar content increased. In terms of flavonoids, the addition of BCP resulted in the significant (*p* < 0.05) increase in quercetin and rutin while epicatechin content significantly (*p* < 0.05) decreased. Moreover, the content of syringic, vanillic, gallic, *trans*-cinnamic, chlorogenic and ferulic acids increased as a result of BCP addition.

Except catechin hydrate, epicatechin and *trans*-cinnamic acid, which remain at similar levels, the content of all quantified phenolic compounds increased as the addition level of BBP in the myrobalan fruit leather increased as a result of the high content of phenolic compounds in BBP. The most important increase was found in syringic acid (from 0.19 in MFL10 to 2.29 mg/100 g in MFL10BB2). Many previous studies reported high contents of syringic, caffeic, ferulic and gallic acids, epicatechin and quercetin in bilberry fruits and extracts [57,58,59,60,61]. Mikulic-Petkovsek et al. [62] reported high contents of epicatechin (27.9–63.1 mg/kg) in different blackcurrant cultivars while Azman et al. [63] rated caffeic acid, *p*-coumaric and ferulic acids as common hydroxycinnamic acids found in blackcurrant pomace. Blejan et al. [64] found ellagic acid (13.60 mg/g) as the dominant phenolic acid in bilberry pomace, followed by catechin (10.86 mg/g), while in blackcurrant pomace the major phenolic compounds were catechin (12.53 mg/g), epicatechin (3.09 mg/g), chlorogenic acid (4.29 mg/g) and trans-cinnamic acid (1.82 mg/g). Untea et al. [65] found 14.7 mg/100 g epicatechin, 25.3 mg/100 g catechin, 11.3 mg/100 g gallic acid, 5 mg/100 g ferulic acid, 3.5 mg/100 g vanillic acid and only 1 mg/100 g rutin in blackcurrant pomace.

The correlations between the content of phenolic compounds, organic acids, TPC and RDA in the myrobalan leathers were investigated and the results are presented in Table 8.

The data revealed significant (*p* < 0.01) and strong correlations between TPC and RSA, as well as between TPC, RSA and the contents of vanillic acid, gallic acid, caffeic acid and rutin. Significant (*p* < 0.01) and strong correlations were also found between the content of organic acids, as well as between the content of organic acids, epicatechin and catechin hydrate. The ascorbic acid content was poorly correlated with RSA due to the week contribution of ascorbic acid to the antioxidant activity of myrobalan leathers.

### 3.7. Sensory Analysis

**Figure 6:**
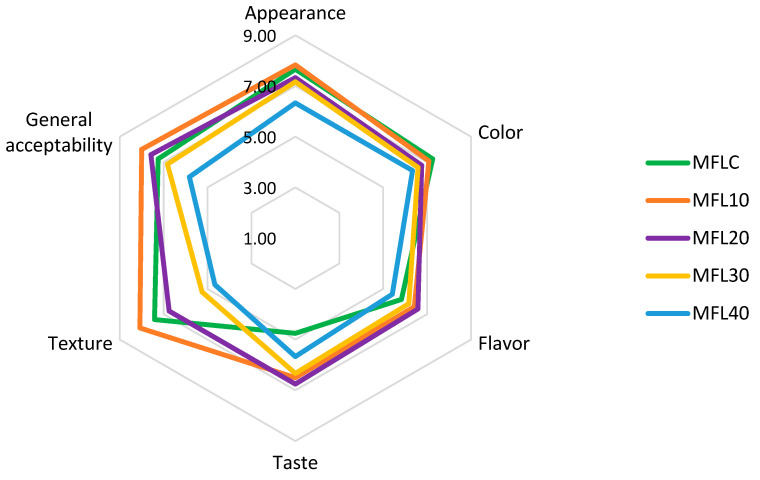
Effect of pulp/sugar ratio on the sensory properties of myrobalan leathers.

**Figure 7:**
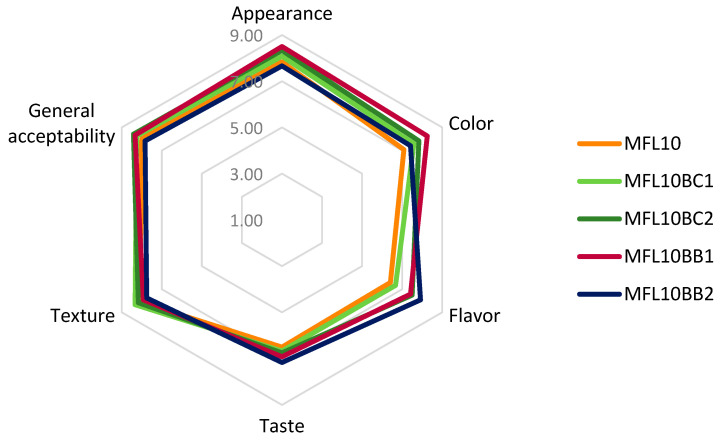
Effect of blackcurrant (BC) and bilberry (BB) pomace powder addition on the sensory properties of myrobalan leathers.

The effects of the pulp/sugar ratio and blackcurrant and bilberry pomace powder addition on the results of the sensory analysis are presented in Figure 6 and Figure 7, respectively. The detailed results of the sensory analysis are presented in Tables S1 and S2 available in the Supplementary Materials.

The increase in sugar level determined the lightening of the color, revealed also by the instrumental color analysis, which was negatively appreciated by the panelists. The texture was seriously affected by the increase in the sugar level, going from an elastic leather texture in control samples to a caramel texture, increasingly soft, crumbly, with increasingly reduced elasticity and greater adhesiveness and stickiness as the sugar level increased.

The control sample (MFLC) had an intense sour taste, confirmed by the high level of titratable acidity and organic acid content. Sugar addition at low levels (90:10 and 80:20) had a positive impact on the taste but also amplified the fruity aroma of the product, which led to an increase in the taste and aroma scores of these samples. Previously, Okilya et al. [66] also reported the enhancement of the fruit leather taste by adding sugar. Higher levels of sugar addition led to a decrease in the ratings for flavor and taste due to the dilution of the fruity taste and aroma and the increase in the sweet taste sensation. Other previous studies found also a decline in the taste rating as a result of the excessive increase in the amount of sugar in fruit leather [44,67]. In terms of overall acceptability, the MFL10 sample was best appreciated, followed by MFL20, without statistically significant differences between them (*p* > 0.05), while MFL40 obtained an overall acceptability score below 6, making it the least-liked fruit leather.

The results of the sensory analysis demonstrated that MGL10 was the best formula for further development of myrobalan leathers with blackcurrant and bilberry pomace addition. The addition of BCP improved the color of the leather, mostly at 2%. The color of the myrobalan leather with addition of bilberry powder was better appreciated at 1% level while 2% addition excessively darkened the fruit leather. Addition of both pomaces improved the flavor and the taste of the myrobalan leather by slightly adding specific fruit flavors. In particular, the bilberry taste intensified with increasing bilberry pomace powder addition. The texture improved at 1% blackcurrant pomace powder addition but 2% addition resulted in a decrease in the ratings for texture due to the increase in leather hardness and adhesiveness. Momchilova et al. [27] also noticed an increase in adhesiveness of the plum leather as a result of the enrichment with chokeberry and raspberry pressings. As previously reported [24], the increase in adhesiveness decreased the acceptance of the fruit leather. The overall score for sensory attributes was considerably improved by the addition of 2% blackcurrant pomace powder or 1% bilberry pomace powder.

## 4. Conclusions

This study proposes myrobalan plum (*Prunus cerasifera*), a widespread but underutilized fruit, rich in dietary fiber, organic acids and bioactive compounds, and blackcurrant or bilberry pomace, valuable by-products typically discarded as waste, to be manufactured into a fruit leather, a nutritious and functional snack, with attractive sensory attributes and health benefits. The increasing level of sugar in the leather formulation resulted in a progressive increase in lightness and decrease in the a and b color values as compared to the control, the color difference becoming “perceptible at a glance” over 20% sugar content. The texture was also affected by the increase in the sugar level, going from an elastic leather texture in control samples to a caramel texture, increasingly soft and crumbly. Moreover, increasing sugar content decreased acidity and antioxidant content in myrobalan leathers. The myrobalan plum leather prepared from 90% pulp and 10% sugar was selected as the best formula for further development of myrobalan plum leathers. Addition of BCP and BBP improved the color, taste and flavor and significantly increased the phenolic content and antioxidant activity of the myrobalan plum leathers. Due to their attractive palatability and high bioactive content, the newly developed fruit leathers could be recommended as functional snack foods. Further sensory evaluation by a larger panel, packaging and long-term storage investigations are needed to assess the commercial potential of the fruit leathers developed in this study. A thorough assessment of economic feasibility and sustainability is essential to improving the market viability of these products.

## Figures and Tables

**Table 1:** Total phenolic content (TPC) and DPPH radical scavenging activity (RSA) in blackcurrant pomace powder, bilberry pomace powder and in the flesh and peel of myrobalan fruits.

	TPC (mg GAE/100 g)	RSA (mmol Trolox/100 g)
Myrobalan fruit flesh	85.64 ± 2.77	0.82 ± 0.01
Myrobalan fruit peel	138.36 ± 5.78	1.34 ± 0.02
Blackcurrant pomace powder	978.85 ± 17.37	2.48 ± 0.11
Bilberry pomace powder	3456.23 ± 25.65	10.18 ± 0.37

**Table 2:** Effect of the pulp/sugar ratio on color parameters and titratable acidity of myrobalan fruit leathers.

	MFLC	MFL10	MFL20	MFL30	MFL40
L*	39.49 ± 0.42 ^b^	39.95 ± 0.58 ^b^	42.01 ± 0.52 ^a^	41.50 ± 0.46 ^a^	40.11 ± 0.62 ^b^
a*	17.06 ± 0.22 ^a^	16.30 ± 0.39 ^b^	15.20 ± 0.57 ^c^	13.97 ± 0.61 ^d^	12.84 ± 0.19 ^e^
b*	25.89 ± 0.41 ^cd^	26.37 ± 0.33 ^c^	28.08 ± 0.56 ^a^	27.34 ± 0.71 ^b^	25.63 ± 0.57 ^d^
C	31.01 ± 0.45 ^b^	31.00 ± 0.36 ^b^	31.93 ± 0.65 ^a^	30.70 ± 0.90 ^b^	28.63 ± 0.60 ^c^
h	56.62 ± 0.24 ^d^	58.29 ± 0.68 ^c^	61.57 ± 0.83 ^b^	62.94 ± 0.43 ^a^	63.55 ± 0.37 ^a^
ΔE	-	1.28 ± 0.38 ^c^	3.67 ± 0.59 ^b^	3.88 ± 0.43 ^ab^	4.29 ± 0.23 ^a^
BI	35.61 ± 0.24 ^a^	34.49 ± 0.88 ^b^	31.63 ± 1.06 ^c^	29.88 ± 1.10 ^d^	28.59 ± 0.45 ^e^
Titratable acidity (g MA/100 g)	10.98 ± 0.28 ^a^	6.73 ± 0.05 ^b^	4.89 ± 0.09 ^c^	3.32 ± 0.05 ^d^	2.41 ± 0.09 ^e^
Thickness (mm)	0.95 ± 0.13 ^d^	1.11 ± 0.15 ^c^	1.33 ± 0.17 ^b^	1.58 ± 0.15 ^a^	1.69 ± 0.18 ^a^

Different lowercase letters indicate significant differences between fruit leather formulations (*p* < 0.05).

**Table 3:** Effect of blackcurrant (BCP) and bilberry (BBP) pomace powder addition on color and titratable acidity of myrobalan leathers (pulp/sugar ratio = 90:10).

	MFL10	MFL10BC1	MFL10BC2	MFL10BB1	MFL10BB2
L*	39.95 ± 0.58 ^a^	34.15 ± 2.10 ^b^	30.15 ± 0.31 ^c^	20.90 ± 0.15 ^d^	19.24 ± 0.39 ^e^
a*	16.30 ± 0.39 ^a^	15.52 ± 0.35 ^b^	13.85 ± 0.09 ^c^	5.56 ± 0.21 ^d^	4.25 ± 0.25 ^e^
b*	26.37 ± 0.33 ^a^	18.96 ± 0.46 ^b^	15.60 ± 0.30 ^c^	9.67 ± 0.22 ^d^	5.91 ± 0.53 ^e^
C	31.00 ± 0.36 ^a^	24.50 ± 0.54 ^b^	20.89 ± 0.21 ^c^	11.16 ± 0.18 ^d^	7.36 ± 0.62 ^e^
h	58.29 ± 0.68 ^b^	50.69 ± 0.49 ^d^	48.46 ± 0.44 ^e^	60.13 ± 1.25 ^a^	54.60 ± 1.88 ^c^
ΔE	-	9.58 ± 0.93 ^d^	14.78 ± 0.60 ^c^	27.52 ± 0.57 ^b^	31.51 ± 0.69 ^a^
BI	34.49 ± 0.88 ^b^	36.33 ± 2.05 ^a^	36.13 ± 0.50 ^a^	23.10 ± 0.49 ^c^	18.46 ± 1.23 ^d^
Titratable acidity (g MA/100 g)	6.73 ± 0.15 ^c^	6.88 ± 0.21 ^bc^	7.00 ± 0.09 ^bc^	7.12 ± 0.14 ^ab^	7.33 ± 0.18 ^a^
Thickness (mm)	1.11 ± 0.15 ^c^	1.37 ± 0.14 ^a^	1.30 ± 0.13 ^ab^	1.26 ± 0.12 ^ab^	1.19 ± 0.17 ^bc^

Different lowercase letters indicate significant differences between fruit leather formulations (*p* < 0.05).

**Table 4:** Effect of the pulp/sugar ratio on the organic acid content of myrobalan leathers.

	MFLC	MFL10	MFL20	MFL30	MFL40
Malic acid (g/100 g)	7.80 ± 0.27 ^a^	4.84 ± 0.19 ^b^	3.36 ± 0.11 ^c^	2.32 ± 0.08 ^d^	1.72 ± 0.06 ^e^
Tartaric acid (g/100 g)	1.26 ± 0.05 ^a^	0.75 ± 0.03 ^b^	0.50 ± 0.02 ^c^	0.30 ± 0.01 ^d^	0.22 ± 0.01 ^e^
Citric acid (g/100 g)	1.38 ± 0.05 ^a^	0.88 ± 0.03 ^b^	0.64 ± 0.03 ^c^	0.46 ± 0.02 ^d^	0.37 ± 0.01 ^e^
Oxalic acid (g/100 g)	2.58 ± 0.08 ^a^	1.89 ± 0.06 ^b^	1.30 ± 0.04 ^c^	0.99 ± 0.04 ^d^	0.81 ± 0.03 ^e^
Ascorbic acid (mg/100 g)	26.06 ± 0.81 ^a^	16.55 ± 0.63 ^b^	10.37 ± 0.55 ^c^	6.79 ± 0.33 ^d^	3.95 ± 0.42 ^e^

Different lowercase letters indicate significant differences between fruit leather formulations (*p* < 0.05).

**Table 5:** Effect of blackcurrant (BCP) and bilberry (BBP) pomace powder addition on the organic acid content of myrobalan leathers.

	MFL10	MFL10BC1	MFL10BC2	MFL10BB1	MFL10BB2
Malic acid (g/100 g)	4.84 ± 0.14 ^a^	4.88 ± 0.12 ^a^	4.82 ± 0.09 ^a^	4.90 ± 0.15 ^a^	4.74 ± 0.14 ^a^
Tartaric acid (g/100 g)	0.75 ± 0.03 ^a^	0.73 ± 0.03 ^a^	0.71 ± 0.02 ^ab^	0.67 ± 0.02 ^bc^	0.65 ± 0.02 ^c^
Citric acid (g/100 g)	0.88 ± 0.04 ^c^	1.03 ± 0.05 ^ab^	1.10 ± 0.04 ^a^	0.85 ± 0.03 ^c^	0.98 ± 0.04 ^b^
Oxalic acid (g/100 g)	1.89 ± 0.06 ^a^	1.68 ± 0.05 ^b^	1.63 ± 0.07 ^b^	1.66 ± 0.04 ^b^	1.65 ± 0.05 ^b^
Ascorbic acid (mg/100 g)	16.55 ± 0.72 ^a^	17.90 ± 0.66 ^a^	19.71 ± 0.65 ^a^	19.88 ± 0.44 ^a^	22.78 ± 0.65 ^a^

Different lowercase letters indicate significant differences between fruit leather formulations (*p* < 0.05).

**Table 6:** Effect of the pulp/sugar ratio on the phenolic content of myrobalan leathers.

	MFLC	MFL10	MFL20	MFL30	MFL40
Vanillic acid	0.27 ± 0.02 ^a^	0.13 ± 0.01 ^b^	0.08 ± 0.01 ^c^	0.05 ± 0.01 ^d^	0.02 ± 0.01 ^e^
Rutin	0.26 ± 0.02 ^a^	0.24 ± 0.01 ^a^	0.18 ± 0.01 ^b^	0.13 ± 0.01 ^c^	0.09 ± 0.01 ^d^
Quercetin	0.17 ± 0.01 ^a^	0.15 ± 0.01 ^b^	0.11 ± 0.01 ^c^	0.09 ± 0.01 ^d^	0.04 ± 0.01 ^e^
Gallic acid	0.13 ± 0.01 ^a^	0.09 ± 0.01 ^b^	0.05 ± 0.01 ^c^	0.03 ± 0.00 ^d^	0.02 ± 0.01 ^d^
Catechin hydrate	1.15 ± 0.05 ^a^	0.87 ± 0.04 ^b^	0.66 ± 0.04 ^c^	0.45 ± 0.01 ^d^	0.17 ± 0.01 ^e^
Syringic acid	0.37 ± 0.13 ^a^	0.19 ± 0.01 ^b^	0.16 ± 0.02 ^bc^	0.08 ± 0.01 ^cd^	0.03 ± 0.01 ^d^
Epicatechin	5.37 ± 0.21 ^a^	3.96 ± 0.16 ^b^	3.26 ± 0.12 ^c^	1.78 ± 0.07 ^d^	0.87 ± 0.06 ^e^
*Trans*-cinnamic acid	0.06 ± 0.01 ^a^	0.05 ± 0.01 ^a^	0.03 ± 0.00 ^b^	0.02 ± 0.01 ^b^	nd
Chlorogenic acid	0.47 ± 0.02 ^a^	0.36 ± 0.02 ^b^	0.23 ± 0.01 ^c^	0.18 ± 0.01 ^d^	0.12 ± 0.01 ^e^
Caffeic acid	0.06 ± 0.01 ^a^	0.06 ± 0.01 ^a^	0.04 ± 0.01 ^b^	nd	nd
*p*-Coumaric acid	0.21 ± 0.01 ^a^	0.17 ± 0.01 ^b^	0.15 ± 0.01 ^bc^	0.12 ± 0.01 ^cd^	0.09 ± 0.04 ^d^
Ferulic acid	0.11 ± 0.01 ^a^	0.09 ± 0.00 ^b^	0.07 ± 0.01 ^c^	0.05 ± 0.01 ^d^	nd

Different lowercase letters indicate significant differences between fruit leather formulations (*p* < 0.05); nd—not detected.

**Table 7:** Effect of blackcurrant (BCP) and bilberry (BBP) pomace powder addition on the phenolic content of myrobalan leathers.

	MFL10	MFL10BC1	MFL10BC2	MFL10BB1	MFL10BB2
Vanillic acid	0.13 ± 0.01 ^e^	0.27 ± 0.02 ^d^	0.36 ± 0.02 ^c^	0.43 ± 0.02 ^b^	0.76 ± 0.03 ^a^
Rutin	0.24 ± 0.01 ^e^	0.29 ± 0.02 ^d^	0.44 ± 0.02 ^c^	0.53 ± 0.03 ^b^	0.94 ± 0.03 ^a^
Quercetin	0.15 ± 0.01 ^e^	0.64 ± 0.03 ^b^	1.06 ± 0.05 ^a^	0.28 ± 0.02 ^d^	0.55 ± 0.03 ^c^
Gallic acid	0.09 ± 0.01 ^d^	0.12 ± 0.01 ^d^	0.23 ± 0.02 ^c^	0.31 ± 0.02 ^b^	0.75 ± 0.04 ^a^
Catechin hydrate	0.87 ± 0.04 ^ab^	0.85 ± 0.03 ^b^	0.93 ± 0.05 ^a^	0.83 ± 0.04 ^b^	0.93 ± 0.04 ^a^
Syringic acid	0.19 ± 0.01 ^e^	1.53 ± 0.08 ^c^	2.65 ± 0.11 ^a^	1.13 ± 0.05 ^d^	2.29 ± 0.09 ^b^
Epicatechin	3.96 ± 0.16 ^a^	3.23 ± 0.14 ^b^	3.25 ± 0.12 ^b^	3.58 ± 0.15 ^a^	3.72 ± 0.16 ^a^
*Trans*-cinnamic acid	0.05 ± 0.01 ^c^	0.30 ± 0.02 ^b^	0.55 ± 0.03 ^a^	0.05 ± 0.01 ^c^	0.07 ± 0.01 ^c^
Chlorogenic acid	0.36 ± 0.02 ^d^	0.47 ± 0.02 ^c^	0.68 ± 0.03 ^b^	0.39 ± 0.02 ^d^	0.83 ± 0.04 ^a^
Caffeic acid	0.06 ± 0.02 ^c^	0.04 ± 0.01 ^c^	0.06 ± 0.00 ^c^	0.24 ± 0.01 ^b^	0.42 ± 0.03 ^a^
*p*-Coumaric acid	0.17 ± 0.01 ^c^	0.14 ± 0.01 ^d^	0.19 ± 0.01 ^c^	0.22 ± 0.02 ^b^	0.32 ± 0.02 ^a^
Ferulic acid	0.09 ± 0.01 ^d^	0.26 ± 0.02 ^b^	0.42 ± 0.03 ^a^	0.16 ± 0.01 ^c^	0.40 ± 0.02 ^a^

Different lowercase letters indicate significant differences between fruit leather formulations (*p* < 0.05).

**Table 8:** Correlation coefficients between phenolic compounds, organic acids, total phenolic content (TPC) and DPPH radical scavenging activity (RSA) in myrobalan leathers.

	Rutin	Quercetin	Gallic Acid	Catechin Hydrate	Syringic Acid	Epicatechin	*Trans*-Cinnamic Acid	Chlorogenic Acid	Caffeic Acid	Coumaric Acid	Ferulic Acid	Malic Acid	Tartaric Acid	Citric Acid	Oxalic Acid	Ascorbic Acid	TPC	RSA
Vanillic acid	0.98 **	0.55	0.96 **	0.59	0.79 *	0.48	0.23	0.90 **	0.90 **	0.93 **	0.80 **	0.47	0.37	0.55	0.32	0.44	0.95 **	0.94 **
Rutin	1	0.51	0.98 **	0.51	0.76 *	0.39	0.17	0.86 **	0.94 **	0.92 **	0.77 *	0.34	0.24	0.42	0.21	0.31	0.97 **	0.96 **
Quercetin		1	0.43	0.46	0.93 **	0.21	0.93 **	0.77 *	0.22	0.36	0.93 **	0.29	0.24	0.52	0.13	0.36	0.36	0.30
Gallic acid			1	0.43	0.70 *	0.33	0.07	0.82 **	0.96 **	0.91 **	0.72 *	0.28	0.19	0.35	0.16	0.24	0.97 **	0.97 **
Catechin hydrate				1	0.49	0.95 **	0.32	0.73 *	0.34	0.70 *	0.52	0.94 **	0.91 **	0.95 **	0.88 **	0.95 **	0.55	0.41
Syringic acid					1	0.25	0.75 *	0.89 **	0.54	0.61	0.98 **	0.30	0.22	0.51	0.13	0.34	0.64	0.60
Epicatechin						1	0.10	0.56	0.29	0.66 *	0.29	0.95 **	0.94 **	0.89 **	0.93 **	0.94 **	0.48	0.35
*Trans*-cinnamic acid							1	0.52	0.01	0.05	0.73 *	0.21	0.19	0.44	0.09	0.31	0.02	−0.05
Chlorogenic acid								1	0.67 *	0.84 **	0.92 **	0.57	0.51	0.72 *	0.45	0.59	0.81 **	0.73 *
Caffeic acid									1	0.88 **	0.54	0.21	0.12	0.23	0.12	0.16	0.96 **	0.99 **
Coumaric acid										1	0.64 *	0.59	0.52	0.61	0.51	0.55	0.95 **	0.91 **
Ferulic acid											1	0.32	0.26	0.54	0.16	0.37	0.66	0.61
Malic acid												1	0.99 **	0.96 **	0.97 **	0.99 **	0.44	0.29
Tartaric acid													1	0.94 **	0.98 **	0.98 **	0.35	0.19
Citric acid														1	0.90 **	0.97 **	0.47	0.31
Oxalic acid															1	0.96 **	0.33	0.17
Ascorbic acid																1	0.39	0.23
TPC																	1	0.98 **
RSA																		1

* *p* < 0.05; ** *p* < 0.01.

## Data Availability

The original contributions presented in this study are included in the article. Further inquiries can be directed to the corresponding author.

## Supplementary Materials

The following supporting information can be downloaded at: https://www.mdpi.com/article/10.3390/foods14203457/s1, Table S1. Effect of pulp:sugar ratio on the sensory properties of myrobalan leathers. Table S2. Effect of blackcurrant (BC) and bilberry (BB) pomace powder addition on the sensory properties of myrobalan leathers (pulp:sugar ratio = 90:10).
