# Supplementary material for: Physicochemical, Phytochemical and Sensory Properties of Myrobalan (Prunus cerasifera L.) Fruit Leather: Effects of Sugar Concentration and Enrichment with Blackcurrant and Bilberry Pomace Powders
Source: Foods. 2025 Oct 10;14(20):3457. doi: 10.3390/foods14203457 (PMC12563754; doi:10.3390/foods14203457)
Supplement: Supplementary file 1 [file foods-14-03457-s001.zip › foods-3913039-supplementary.pdf]

Table S1. Effect of pulp:sugar ratio on the sensory properties of myrobalan leathers.

	MFLC	MFL10	MFL20	MFL30	MFL40
Appearance	7.67 ± 0.78^{ab}	7.83 ± 0.58^{a}	7.33 ± 0.65^{ab}	7.17 ± 0.72^{b}	6.33 ± 0.65^{c}
Color	7.25 ± 0.62^{a}	7.08 ± 0.79^{ab}	6.75 ± 0.75^{abc}	6.58 ± 0.51^{bc}	6.33 ± 0.49^{c}
Flavor	5.83 ± 0.58^{bc}	6.42 ± 0.51^{a}	6.58 ± 0.51^{a}	6.17 ± 0.58^{ab}	5.42 ± 0.51^{c}
Taste	4.75 ± 0.75^{c}	6.50 ± 0.52^{a}	6.75 ± 0.45^{a}	6.33 ± 0.49^{a}	5.67 ± 0.49^{b}
Texture	7.42 ± 0.51^{b}	8.08 ± 0.67^{a}	6.75 ± 0.45^{c}	5.25 ± 0.75^{d}	4.67 ± 0.49^{e}
General acceptability	7.25 ± 0.45^{bc}	8.00 ± 0.74^{a}	7.58 ± 0.51^{ab}	6.83 ± 0.39^{c}	5.83 ± 0.94^{d}

Different lowercase letters indicate significant differences between fruit leather formulations ($p < 0.05$).

Table S2. Effect of blackcurrant (BC) and bilberry (BB) pomace powder addition on the sensory properties of myrobalan leathers (pulp:sugar ratio = 90:10).

	MFL10	MFL10BC1	MFL10BC2	MFL10BB1	MFL10BB2
Appearance	7.83 ± 0.58^{cd}	8.08 ± 0.29^{bc}	8.33 ± 0.49^{ab}	8.50 ± 0.52^{a}	7.67 ± 0.49^{d}
Color	7.08 ± 0.79^{c}	7.58 ± 0.51^{b}	7.83 ± 0.39^{ab}	8.25 ± 0.45^{a}	7.42 ± 0.51^{bc}
Flavor	6.42 ± 0.51^{b}	6.67 ± 0.49^{b}	7.50 ± 0.52^{a}	7.42 ± 0.36^{a}	7.92 ± 0.79^{a}
Taste	6.50 ± 0.52^{c}	6.67 ± 0.49^{bc}	6.75 ± 0.45^{bc}	6.92 ± 0.51^{ab}	7.17 ± 0.39^{a}
Texture	8.08 ± 0.67^{ab}	8.33 ± 0.49^{a}	8.17 ± 0.58^{ab}	7.92 ± 0.67^{ab}	7.75 ± 0.62^{b}
General acceptability	8.00 ± 0.74^{ab}	8.25 ± 0.45^{ab}	8.42 ± 0.51^{a}	8.33 ± 0.49^{a}	7.83 ± 0.58^{b}

Different lowercase letters indicate significant differences between fruit leather formulations ($p < 0.05$).
